# Prevalence and risk factors of helminths and intestinal protozoa infections among children from primary schools in western Tajikistan

**DOI:** 10.1186/1756-3305-4-195

**Published:** 2011-10-07

**Authors:** Barbara Matthys, Mohion Bobieva, Gulzira Karimova, Zulfira Mengliboeva, Vreni Jean-Richard, Malika Hoimnazarova, Matluba Kurbonova, Laurent K Lohourignon, Jürg Utzinger, Kaspar Wyss

**Affiliations:** 1 Swiss Centre for International Health, Swiss Tropical and Public Health Institute, P.O. Box, CH-4002 Basel, Switzerland; 2 University of Basel, P.O. Box, CH-4003 Basel, Switzerland; 3 Republican Tropical Diseases Centre of the Republic of Tajikistan, Str. A. Dehoti 48, 734029 Dushanbe, Tajikistan; 4 Project Sino, House No 1, 5th drive, Rudaki Avenue, 734001 Dushanbe, Tajikistan; 5 Department of Epidemiology and Public Health, Swiss Tropical and Public Health Institute, P.O. Box, CH-4002 Basel, Switzerland; 6 UFR Biosciences, Université de Cocody, 22 PB 770, Abidjan 22, Côte d'Ivoire

## Abstract

**Background:**

Intestinal parasitic infections represent a public health problem in Tajikistan, but epidemiological evidence is scarce. The present study aimed at assessing the extent of helminths and intestinal protozoa infections among children of 10 schools in four districts of Tajikistan, and to make recommendations for control.

**Methods:**

A cross-sectional survey was carried out in early 2009. All children attending grades 2 and 3 (age: 7-11 years) from 10 randomly selected schools were invited to provide a stool sample and interviewed about sanitary situation and hygiene behaviour. A questionnaire pertaining to demographic and socioeconomic characteristics was addressed to the heads of households. On the spot, stool samples were subjected to duplicate Kato-Katz thick smear examination for helminth diagnosis. Additionally, 1-2 g of stool was fixed in sodium acetate-acetic acid-formalin, transferred to a specialised laboratory in Europe and examined for helminths and intestinal protozoa. The composite results from both methods served as diagnostic 'gold' standard.

**Results:**

Out of 623 registered children, 602 participated in our survey. The overall prevalence of infection with helminths and pathogenic intestinal protozoa was 32.0% and 47.1%, respectively. There was pronounced spatial heterogeneity. The most common helminth species was *Hymenolepis nana *(25.8%), whereas the prevalences of *Ascaris lumbricoides*, hookworm and *Enterobius vermicularis *were below 5%. The prevalence of pathogenic intestinal protozoa, namely *Giardia intestinalis *and *Entamoeba histolytica/E. dispar *was 26.4% and 25.9%, respectively. Almost half of the households draw drinking water from unimproved sources, such as irrigation canals, rivers and unprotected wells. Sanitary facilities were pit latrines, mostly private, and a few shared with neighbours. The use of public tap/standpipe as a source of drinking water emerged as a protective factor for *G. intestinalis *infection. Protected spring water reduced the risk of infection with *E. histolytica/E. dispar *and *H. nana*.

**Conclusions:**

Our data obtained from the ecological 'lowland' areas in Tajikistan call for school-based deworming (recommended drugs: albendazole and metronidazole), combined with hygiene promotion and improved sanitation. Further investigations are needed to determine whether *H. nana *represents a public health problem.

## Background

Infections with helminths (e.g. *Ascaris lumbricoides*, hookworm, *Hymenolepis nana *and *Trichuris trichiura*) and intestinal protozoa (e.g. the pathogenic *Entamoeba histolytica *and *Giardia intestinalis*) are closely linked with conditions of poverty, unsafe water, sanitation and hygiene [1]. More than 2 billion people might be infected with helminths, mainly in the developing world [2]. At highest risk of morbidity are pre-school and school-aged children and pregnant women [3]. Negative effects of helminth infections include diminished physical fitness and growth retardation, and delayed intellectual development and cognition [2,3]. Vitamin A deficiency, malabsorption of vitamin B_12 _and fat and nutritional deficiencies in children might be associated with *G. intestinalis*, which may lead to serious organ damage [4]. Morbidity due to *E. histolytica *includes diarrhoea and dysentery in children and liver abscess in severe cases [5].

It is widely acknowledged that helminthiasis and intestinal protozoa infections are of considerable public health importance in Tajikistan and elsewhere in Central Asia [6,7], but the geographical distribution and regional burden remain to be determined. Previous research has mainly focussed on parasitic diseases of livestock and most of the available literature is in Russian. Recently, the World Health Organization (WHO) presented a simple methodology to assess the prevalence of helminths, stratified by ecozones, for settings where information is scarce [8]. Once high-risk areas are identified (e.g. > 20% of school-aged children infected with soil-transmitted helminths), WHO recommends deworming of all school-aged children at least once every year [9]. Whenever resources allow, deworming should be complemented with improved access to safe drinking water and sanitation, health education and hygiene behaviour change, coupled with regular monitoring and surveillance. Several countries have launched their helminthiasis control programmes and made progress towards achieving deworming coverage rates of 75% of school-aged children [10].

The Swiss Health Reform and Family Medicine Support Project (Project Sino in short) in Tajikistan contributes to the national health sector reform programme. The project aims to improve the population's health status and access to health services, particularly for poor groups. Among other issues, the project developed an accessible and sustainable family medicine model that is affordable by local communities as shown in pilot districts. The project initiates further evidence-based activities and encourages operational research at the interface of family medicine services and communities with an emphasis on reducing the burden of diseases that are of public health importance [11-16]. The aim of the present study was to assess the prevalence of helminths and intestinal protozoa infections among school-aged children in four districts of Project Sino, and to make recommendations for control.

## Materials and methods

### Study area and context

Tajikistan is a mountainous land-locked country in Central Asia with approximately 7 million inhabitants, most of whom live in rural areas (73.7% in 2009) [17,18]. In 2010, the *per capita *gross development product (GDP) was US$ 2, 000, and hence Tajikistan ranked at position 190 out of 228 countries included in the list of the CIA world factbook [19]. Even though the national economy has grown considerably in the past several years, two-thirds of the population still live on less than US$ 2.15 per day. Agriculture remains the primary sector of the national economy, contributing 24% of the national GDP and 66% of employment. Remittances are a vital source of income for many Tajik households, facilitated through working in the construction sector in Russia. Labour migrants are primarily young men from rural areas [20].

The regional climate is continental, close to Mediterranean with dominant spring-winter precipitation, hot and dry summers and cold winters [21]. Water is becoming increasingly scarce due to rapid shrinkage of glaciers, conflicts with neighbouring downstream countries on water provision used for irrigation purposes (for cotton and to a lesser extent rice cultivation), and deterioration of irrigation and drainage systems [20].

**Figure 1:**
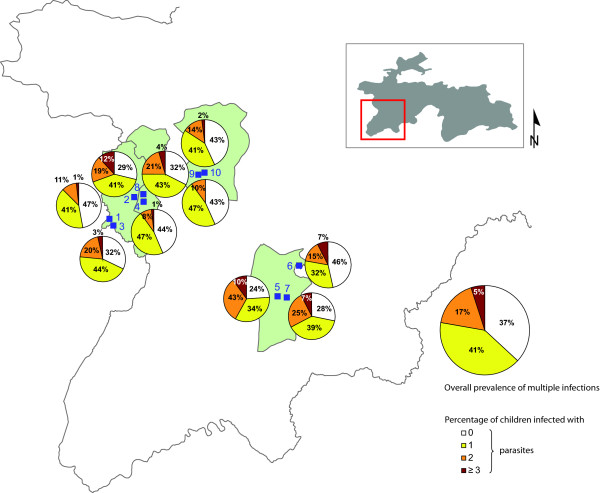
**Prevalence of multiple species infection with helminths and pathogenic intestinal protozoa, stratified by school, in western Tajikistan in early 2009**.

Our study was carried out in four districts of Project Sino located in the western part of Tajikistan in early 2009 (Figure 1). Prior to our survey, relevant literature considering the local context of Tajikistan (e.g. peer-reviewed articles obtained from searching electronic databases such as PubMed and ISI Web of Knowledge) and reports and national statistics from WHOLIS and the WHO regional office in Europe were reviewed.

### Selection of study population

Schoolchildren attending grades 2 and 3 (age: 7-11 years) were chosen according to WHO recommendations [22]. Schools are a convenient platform to conduct surveys and schoolchildren are at high risk of infections with helminths and other intestinal parasites. Selection of a relatively narrow age range results in smaller confidence intervals around point prevalence estimates. School enrolment rates are high in Tajikistan, i.e. 97% primary net school enrolment in 2005-2009 [23]. We adhered to a rapid appraisal methodology proposed by WHO, suggesting a minimum of 50 schoolchildren to be examined per school [8,9].

### Study design and selection of schools

In a first step, the heads of educational departments from all four districts were asked by a Project Sino representative for community outreach activities in January 2009 to prepare a list of all primary schools in their respective district, including the number of children attending grades 2 and 3. A total number of 300 primary schools were listed in these districts. In 143 of these schools, less than 50 children attended grades 2 and 3, and hence these were excluded. Another 10 schools from one district were excluded because of recent deworming activities implemented by a non-governmental organization (NGO). From the remaining 147 schools, 10 were selected by means of a simple random sampling procedure. In each school, 60-70 children were selected (allowing for drop-outs to gather data from at least 50 children per school), and hence obtaining a minimal sample size of 500 fully complying children.

### Field procedures

School directors from the selected schools and teachers of grades 2 and 3 were visited by the survey team 1 week prior to our cross-sectional parasitological and questionnaire surveys. A written informed consent form for the parents/guardians of participating children, a questionnaire for the household heads, and a small plastic container for collection of stool samples were left with the teachers and distributed to eligible children. The questionnaires were pre-tested in a village near Dushanbe, the capital of Tajikistan, and adapted prior to administration.

During the school-based survey, the signed informed consent sheets, household questionnaires and stool samples were collected. Unique identification numbers were assigned to each participating child. A short interview was held with each child, using a questionnaire pertaining to hygiene behaviour, drinking water and sanitation adapted from a standard tool provided by the joint monitoring programme (JPM) of WHO and UNICEF [24]. Each child was weighed to the nearest kg and measured to the nearest cm. At the end of the survey, each child was given a piece of soap and a small pack of iodine-fortified salt as a small token for their participation.

### Laboratory procedures

From each stool sample, duplicate Kato-Katz thick smears were prepared on microscope slides shortly after stool collection by two experienced laboratory technicians from the Republican Tropical Disease Centre (RTDC) [25]. Thick smears were allowed to clear for 45-60 min prior to examination under a light microscope for helminth eggs. The number of helminth eggs was counted and recorded for each species separately. The slides were read on the spot and the teachers and directors were informed about the overall prevalence of helminth infections in their school.

In addition, approximately 1-2 g of stool was placed in a Falcon tube filled with 15 ml of sodium acetate-acetic acid-formalin (SAF) [26,27]. These SAF-fixed stool samples were transferred to a specialised laboratory in Italy and examined there by an experienced laboratory technician for the presence of helminths and intestinal protozoa using an ether-concentration technique, adhering to a standard protocol [28].

For quality control purposes, 10% of the Kato-Katz thick smears were randomly selected and read retrospectively by an experienced laboratory technician in Côte d'Ivoire. A senior laboratory technician from Switzerland checked approximately 5% of the SAF-fixed stool samples that were examined by the ether-concentration method. In case of discordant results, the slides were re-read and results discussed until agreement between the technicians was reached.

### Ethical considerations

The study was approved by the Ministry of Health (MoH) of Tajikistan (reference no. 16/75-92). The study protocol was presented to the Deputy MoH and methodological issues related to the survey were discussed with the heads of collaborating partner institutes (RTDC and State Sanitary Hygiene Surveillance Department). The primary health care network managers of each district health centre and the administrative authorities at community level were informed about the study and their consent was obtained. Parents/guardians of participating children signed a written informed consent prior to study enrolment. Participation was voluntary and children were free to withdraw at any time. At the end of the study, each child was offered an anthelminthic treatment (single oral dose of 400 mg albendazole) free of charge [29]. A feedback session for key stakeholders (e.g. representatives from the MoH and collaboration institutions) was held at the end of the survey to present and discuss the findings and to jointly draft a plan of action.

### Statistical analysis

Data were entered into EpiData version 3.1 (EpiData Association; Odense, Denmark) and internal consistency checks were done. Stata version 10 (Stata Corporation; College Station, TX, USA) was employed for statistical analysis. The children's socioeconomic status was determined using a household-based asset approach that was adapted from previous studies in Tajikistan [11,14]. In brief, a wealth index was constructed by estimating household asset weights by means of a principal component analysis (PCA) methodology [30]. Nine variables were included in the PCA (i.e. frequency of meat consumption, refrigerator, radio, colour television, satellite, DVD recorder, car, mobile phone and fixed line phone). Missing values were replaced with the mean for the corresponding variable [31].

Sources of drinking water were grouped into 'improved' (i.e. piped water into dwelling/yard, public tap or standpipe, protected dug well/spring, bottle water and rainwater) and 'unimproved' sources (i.e. unprotected spring, cart with small tank, tanker truck and surface water) according to a classification used by the JPM of WHO and UNICEF [24].

With regard to the parasitological data, only children who had duplicate Kato-Katz thick smear readings plus results from the ether-concentration test were included in the final analysis. A helminth infection was defined as the presence of at least one helminth egg in one of the two Kato-Katz thick smears and/or the SAF-fixed stool sample. The presence of an intestinal protozoon cyst in the SAF-fixed stool sample subjected to an ether-concentration method was used as our diagnostic approach for these parasites. For helminths, infection intensity at the unit of an individual was determined as the arithmetic mean egg count from two Kato-Katz thick smears, multiplied by a factor 24 to obtain eggs per gram of stool (EPG). Helminth infection intensities were grouped into light (*A. lumbricoides*, 1-4, 999 EPG; *H. nana*, 1-1, 999 EPG; *T. trichiura*, 1-999 EPG); moderate (*A. lumbricoides*, 5, 000-49, 999; *H. nana*, 2, 000-9, 999 EPG; *T. trichiura*, 1, 000-9, 999 EPG); and heavy (*A. lumbricoides*, ≥50, 000 EPG; *H. nana *and *T. trichiura*, ≥10, 000 EPG) [29,32]. No hookworm eggs were found in the Kato-Katz thick smears. For *Enterobius vermicularis*, no attempt was made to estimate infection intensity, because the Kato-Katz technique lacks diagnostic accuracy for this helminth species [33]. Children were grouped into three age classes: (i) 7-8 years; (ii) 9 years; and (iii) 10-11 years.

Proportions were compared using Pearson's χ^2 ^and Fisher's exact test as appropriate. Medians between groups were compared using the Student's *t*-test and Bartlett's test for equal variances, as appropriate. Risk factors for infection with *H. nana*, *G. intestinalis *and *E. histolytica/E. dispar *were analysed by fitting bi- and multivariate logistic regression models. Explanatory variables associated with infection and a *P-*value ≤0.15 were included into a multivariate logistic regression model. A stepwise backward elimination approach removing covariates above a level of 0.15 one after another was employed. Variations of conditions between schools were considered by introducing a school-level random effect. For all tests, 95% confidence intervals (CIs) were calculated.

## Results

### Study compliance

From a total of 623 children registered in grades 2 and 3 in the 10 selected schools, 602 children participated in the cross-sectional survey, owing to an overall compliance of 96.6%. Reasons for non-compliance were absence due to recent travels (n = 9), absence of written informed consent (n = 3), feeling unwell (n = 2) or no specific reason (n = 7). Children's age ranged between 7 and 11 years with a mean of 9.1 years. There was a borderline significant age difference between schools (Bartlett's test for equal variances: χ^2 ^= 16.54, degree of freedom (d.f.) = 9, *P *= 0.056). There were slightly more boys than girls (311 *versus *291, *P *= 0.416) with no sex difference between schools (χ^2 ^= 10.47, d.f. = 9, *P *= 0.314).

### Household profiles

One quarter (24.5%) of the variability of the household's socioeconomic status was explained by the first principal component. Greatest weight was given to households possessing a car (0.44), refrigerator (0.42) and DVD recorder (0.36). After standardising the asset weighed variables, households having a satellite (0.79), refrigerator (0.72) and car (0.64) were scored highest, whereas lowest scores were given to households with no colour television (-0.63), no mobile phone (-0.47) and no DVD recorder (-0.44). A wealth index was created for each child by building a total of all household asset scores and assigning accordingly each child into five wealth quintiles. Finally, each child was grouped into three wealth classes (bottom, 40%; middle, 40%; top, 20%) (Table 1).

**Table 1:** Wealth quintiles based on nine household assets for 602 children aged 7-11 years from 10 schools in western Tajikistan, early 2009

	Wealth quintiles (%)			
	
Household asset variable	Total	Bottom 40% (n = 257)	Middle 40% (n = 225)	Top 20% (n = 120)
Meat consumption				
≥5 times a week	10.6	4.3	9.3	26.7
3-4 times a week	16.0	5.5	22.2	26.7
1-2 times a week	47.2	51.0	48.0	37.5
< 1 times a week	26.3	39.3	20.4	9.2
Has a refrigerator	24.6	2.7	22.2	75.8
Has a radio	60.8	40.9	66.2	93.3
Has a colour television	78.9	59.1	92.9	94.2
Has a satellite	12.3	1.6	9.3	40.8
Has a DVD recorder	58.1	33.5	74.7	86.7
Has a car	31.9	3.5	35.6	85.8
Has mobile phone	68.4	46.7	83.6	86.7
Has a fix phone	9.5	2.3	8.4	26.7

Most households comprised between 6 and 10 individuals. Every fifth household was smaller, counting 3-5 individuals. Large households with 11 persons and more accounted for 10% in our study sample. Two-thirds of the household heads were farmers or craftspeople. Regarding educational attainment, every other household head obtained a secondary school-leaving certificate (11 years of school or more), whereas almost every third had a university degree. Only 3% of the household heads reported not having received any education. With few exceptions, all households kept livestock, such as bullocks, cows, donkeys, goats, horses and sheep. In addition, half of the households kept chickens. Meat was consumed, on average, twice a week.

Slightly more than half of the households (53%) had improved drinking water sources, such as protected springs (20%) and public tap/standpipe (18%). The remaining 47% of the households depended on unimproved sources, i.e. surface water from irrigation canals, rivers and streams (38%), unprotected wells and springs and rain water. There was large heterogeneity of unimproved water sources at the unit of school, varying between 2% and 100%. With regard to sanitation, almost all households used pit latrines that are not connected to a sewage system. Three out of four households had their own latrines, whereas the remaining households shared sanitation facilities with their neighbours in the yard. Two households used a public sanitation facility.

### Helminths and intestinal protozoa infections

Overall, 599 of the interviewed children had a single stool sample subjected to duplicate Kato-Katz thick smear reading and 594 of the children had a small portion of stool fixed in SAF that was examined by an ether-concentration technique for helminths and intestinal protozoa. Complete parasitological data were therefore available for a subsample of 594 participants. Table 2 shows that the prevalence of infection with any helminths or pathogenic intestinal protozoa was 32.0% and 47.1%, respectively. The overall prevalence of soil-transmitted helminths was 8.6%. There was no statistically significant sex difference in the prevalence of any of the helminths identified (*P *> 0.05). *H. nana *was the predominant helminth species (25.8%), whereas all other helminths identified showed prevalences below 5%, e.g. *A. lumbricoides *(4.4%), hookworm (3.5%) and *T. trichiura *(1.4%). With regard to age, *A. lumbricoides *showed a statistically significantly higher prevalence in the youngest children (age 7-8 years, prevalence 9.0% *versus *3.8% and 2.2% in 9-year-old and 10- to 11-year-old children; Fisher's exact test, *P *= 0.021).

**Table 2:** Number (%) of schoolchildren infected with helminths and intestinal protozoa in western Tajikistan, early 2009

Parasite	Overall (n = 594)	Boys (n = 307)	Girls (n = 287)	**χ**^**2**^	*P*-value	School no. 1 (n = 64)	School no. 2 (n = 59)	School no. 3 (n = 59)	School no. 4 (n = 62)	School no. 5 (n = 50)	School no. 6 (n = 54)	School no. 7 (n = 67)	School no. 8 (n = 68)	School no. 9 (n = 60)	School no. 10 (n = 51)
Helminth															

*Hymenolepis nana*^a, b^	153 (25.8)	74 (24.1)	79 (27.5)	0.91	0.341	11 (17.2)	19 (32.2)	17 (28.8)	13 (21.0)	19 (38.0)	11 (20.4)	23 (34.3)	23 (33.8)	6 (10.0)	11(21.6)
*Ascaris lumbricoides*^a, b^	26 (4.4)	16 (5.2)	10 (3.5)	NA	0.323	3 (4.7)	0	1 (1.7)	0	8 (16.0)	4 (7.4)	4 (6.0)	4 (5.9)	0	2 (3.9)
Hookworm^a, b^	21 (3.5)	10 (3.3)	11 (3.8)	NA	0.825	0	4 (6.8)	0	0	5 (10.0)	2 (3.7)	3 (4.5)	2 (2.9)	4 (6.7)	1 (2.0)
*Enterobius vermicularis*^a, b^	15 (2.5)	4 (1.3)	11 (3.8)	NA	0.066	2 (3.1)	1 (1.7)	6 (10.2)	1 (1.6)	2 (4.0)	0	2 (3.0)	1 (1.5)	0	0
*Trichuris trichiura*^a, b^	8 (1.4)	2 (0.7)	6 (2.1)	NA	0.164	0	5 (8.5)	0	0	3 (6.0)	0	0	0	0	0
*Fasciola hepatica*^a, b^	3 (0.5)	2 (0.7)	1 (0.4)	NA	0.999	1 (1.6)	1 (1.7)	0	0	0	0	1 (1.5)	0	0	0
*Hymenolepis diminuta*^a^	3 (0.5)	2 (0.7)	1 (0.4)	NA	0.999	0	0	0	0	0	2 (3.7)	0	0	1 (1.7)	0
*Dicrocoelium dendriticum*^a^	1 (0.2)	0	1 (0.3)	NA	0.483	0	0	0	0	0	0	0	0	1 (1.7)	0
Any helminth	190 (32.0)	91 (29.6)	99 (34.5)	1.61	0.205	15 (23.4)	21 (35.6)	22 (37.3)	13 (21.0)	27 (54.0)	14 (25.9)	29 (43.3)	26 (38.2)	11 (18.3)	12 (23.5)

Any soil-transmitted helminth	51 (8.6)	27 (8.8)	24 (0.4)	NA	0.851	3 (4.7)	9 (15.3)	1 (1.7)	0	13 (26.0)	5 (9.3)	7 (10.5)	6 (8.8)	4 (6.7)	3 (5.9)

Intestinal protozoon															

*Entamoeba coli*^c^	390 (65.7)	196 (63.8)	194 (67.6)	0.93	0.336	41 (64.1)	36 (61.0)	47 (79.7)	42 (67.7)	36 (72.0)	38 (70.4)	47 (70.2)	41 (62.3)	31 (51.7)	31 (60.8)
*Giardia intestinalis*^a^	157 (26.4)	82 (26.7)	75 (26.1)	0.03	0.873	7 (10.9)	24 (40.7)	16 (27.1)	17 (27.4)	12 (24.0)	12 (22.2)	22 (32.8)	16 (23.5)	18 (30.0)	13 (25.5)
*Entamoeba histolytica/E. dispar*^a^	154 (25.9)	82 (26.7)	72 (25.1)	0.20	0.652	19 (29.7)	13 (22.0)	16 (27.1)	11 (17.7)	19 (38.0)	14 (25.9)	21 (31.3)	20 (29.4)	10 (16.7)	11 (21.6)
*Blastocystis hominis*^d^	118 (19.9)	63 (20.5)	55 (19.2)	0.17	0.679	12 (18.8)	11 (18.6)	13 (22.0)	11 (17.7)	9 (18.0)	8 (14.8)	15 (22.4)	17 (25.0)	13 (21.7)	9 (17.7)
*Endolimax nana*^c^	118 (19.9)	61 (19.9)	57 (19.9)	0.00	0.998	13 (20.3)	11 (18.6)	18 (30.5)	7 (11.3)	9 (18.0)	7 (13.0)	19 (28.4)	17 (25.0)	8 (13.3)	9 (17.7)
*Iodamoeba bütschlii*^c^	29 (4.9)	13 (4.2)	16 (5.6)	NA	0.455	4 (6.3)	0	6 (10.1)	0	4 (8.0)	6 (11.1)	4 (6.0)	3 (4.4)	0	2 (3.9)
*Entamoeba hartmanni*^c^	28 (4.7)	14 (4.6)	14 (4.9)	NA	0.855	4 (6.3)	3 (5.1)	2 (3.4)	4 (6.5)	4 (8.0)	2 (3.7)	3 (4.5)	3 (4.4)	1 (1.7)	2 (3.9)
*Chilomastix mesnili*^c^	25 (4.2)	15 (4.9)	10 (3.5)	NA	0.677	2 (3.1)	3 (5.1)	2 (3.4)	0	0	8 (14.8)	4 (6.0)	5 (7.4)	1 (1.7)	0
Any intestinal protozoa	497 (83.7)	255 (83.1)	242 (84.3)	0.17	0.678	53 (82.8)	53 (89.8)	54 (91.5)	50 (80.7)	44 (88.0)	46 (85.2)	60 (89.6)	56 (82.4)	42 (70.0)	39 (76.5)
Any pathogenic intestinal protozoa	280 (47.1)	148 (48.2)	132 (46.0)	0.29	0.589	25 (39.1)	33 (55.9)	29 (58.0)	26 (41.9)	29 (58.0)	22 (40.7)	36 (53.7)	33 (48.5)	26 (43.3)	21 (41.2)

Overall infection prevalence with pathogenic intestinal parasites	375 (63.1)	195 (63.5)	180 (62.7)	0.04	0.840	34 (53.1)	42 (71.2)	40 (67.8)	35 (56.5)	38 (76.0)	29 (53.7)	48 (71.6)	46 (67.7)	34 (56.7)	29 (56.9)

^a ^Pathogenic

^b ^Pooled results from duplicate Kato-Katz thick smear readings and ether-concentration test results

^c ^Non-pathogenic

^d ^Suspected pathogenic

NA *P*-value based on Fisher's exact test

With regard to intestinal protozoa, the most common species was the non-pathogenic *Entamoeba coli *(65.7%). The pathogenic protozoa *G. intestinalis *and *E. histolytica/E. dispar *were detected in 26.4% and 25.9% of the children, respectively. The prevalence of the suspected pathogenic protozoon *Blastocystis hominis *was 19.9%. No sex-related differences were found for any of the intestinal protozoa identified.

The prevalence of single and multiple helminths and pathogenic intestinal protozoa species infections are displayed in Figure 1. Overall, 40.9% of all children had a single species infection, whereas 17.3% had a dual species infection and 4.9% harboured at least three intestinal pathogenic parasite species concurrently. There was considerable heterogeneity of overall infection prevalence between schools, ranging from 53.1% to 76.0%. Prevalence of multiple species infection across schools was between 9.7% and 42.0%. The youngest age group (7-8 years) exhibited a slightly higher infection prevalence of multiple species infection than their older counterparts, but the difference was not statistically significant (Fisher's exact, *P *= 0.061).

### Spatial distribution of intestinal parasite infections

The overall prevalence of any intestinal parasites (pathogenic and non-pathogenic) was 88.2%, ranging from 76.7% to 93.2% across schools. Twenty-seven children (4.6%) were infected with helminths only, with prevalences ranging from 1.7% to 7.8% at the unit of the school. More than half of the children were infected with intestinal protozoa only (56.2%, n = 334), with a range from 38.0% to 64.1% in individual schools. A total of 163 children (27.4%) harboured helminths and intestinal protozoa concurrently, between 18.3% and 54.0% at the unit of the school.

Most widespread co-infections were combinations with *H. nana *and *G. intestinalis *(5.2%), followed by *H. nana *and *E. histolytica/E. dispar *(3.9%). The most common triple infection was *H. nana*, *E. histolytica/E. dispar *and *G. intestinalis *(1.5%).

The prevalence of species-specific helminths and intestinal protozoa infections, stratified by school, is given in Table 2. *H. nana *showed highest infection prevalence exceeding 30% in four schools. The highest infection prevalence of *H. nana *(38.0%) occurred in a school where the highest prevalence of *A. lumbricoides *(16.0%) and hookworm (10.0%) were also observed. *T. trichiura *infections were found only in two schools (8.5% and 6.0%). Regarding intestinal protozoa infections, prevalences exceeding 30% were observed in two schools for *G. intestinalis *(40.7% and 32.8%) and in two schools for *E. histolytica/E. dispar *(38.0% and 31.3%). The peak prevalence of *E. histolytica/E. dispar *was observed in the school where the highest helminth infection prevalence was noted.

### Helminth infection intensities

Helminth infection intensities were estimated based on duplicate Kato-Katz thick smears. The overall geometric mean faecal egg count for *H. nana *was 383 EPG (95% CI: 311-471 EPG), for *A. lumbricoides *it was 223 EPG (95% CI: 154-321 EPG), and the respective estimate for *T. trichiura *was 125 EPG (95% CI: 71-222 EPG). All infections were of light intensity according to WHO cut-offs.

There was no statistically significant difference in infection intensity between boys and girls for *H. nana *(two-sample *t*-test, *t *= -0.014, d.f. = 71, *P *= 0.989) and *A. lumbricoides *(*t *= 0.020, d.f. = 19, *P *= 0.984). Infection intensity of *H. nana *decreased with age (ANOVA, Bartlett's test for equal variances, d.f. = 71, 2; χ^2 ^(2) = 11.50, *P *= 0.003). While the geometric mean faecal egg count of *H. nana *for children aged 7-8 years was 460 EPG (95% CI: 278-762 EPG), it was 401 EPG in 9-year-old children (95% CI: 297-541 EPG), and 308 EPG in the oldest age group investigated (95% CI: 210-450 EPG). Age-related differences were also found for *A. lumbricoides *(ANOVA, Bartlett's test for equal variances, d.f. = 18, 2; χ^2 ^(2) = 8.55, *P *= 0.014). The highest geometric mean faecal egg count was observed for 9-year-old children (290 EPG, 95% CI: 120-701 EPG), whereas lower faecal egg counts were observed for younger and older children (7-8 years, mean 215 EPG, 95% CI: 133-348 EPG; 10-11 years, mean 153 EPG, 95% CI: 38-610 EPG).

### Risk factors for intestinal parasites

Table 3 summarises demographic, socioeconomic, hygiene- and drinking water source-related risk factors for an infection with *G. intestinalis*, *E. histolytica/E. dispar *and *H. nana *according to bivariate and multivariate random effects models. Regarding drinking water sources, use of public tap/standpipe (odds ratio (OR) = 0.35, 95% CI: 0.12-1.00) emerged as a protective factor in the bivariate model for infection with *G. intestinalis*. Protected spring water was a protective factor for *E. histolytica/E. dispar *infections in the bi- and multivariate model (OR = 0.52, 95% CI: 0.31-0.88; OR = 0.41, 95% CI: 0.23-0.74, respectively). Moreover, protected spring water emerged as protecting factor for *H. nana *infections in the bi- and multivariate model (OR = 0.42, 95% CI: 0.20-0.88; OR = 0.44, 95% CI: 0.20-0.97).

**Table 3:** Results from bivariate non-random and random effects multivariate logistic regression models for risk factors of specific intestinal parasitic infections among schoolchildren in western Tajikistan, early 2009

Explanatory variable	*Giardia intestinalis*						*Entamoeba histolytica/E. dispar*						*Hymenolepis nana*					
	**Bivariate model**^**a**^			**Multivariate model**^**b**^			**Bivariate model**^**a**^			**Multivariate model**^**b**^			**Bivariate model**^**a**^			**Multivariate model**^**b**^		

	**OR**	**(95% CI)**	**P-value**^**c**^	**OR**	**95% CI**	**P-value**^**c**^	**OR**	**(95% CI)**	**P-value**^**c**^	**OR**	**95% CI**	**P-value**^**c**^	**OR**	**95% CI**	**P-value**^**c**^	**OR**	**95% CI**	**P-value**^**c**^

Demography																		
Sex																		
Male	1.00						1.00						1.00					
Female	0.97	(0.68, 1.40)	0.889				0.91	(0.64, 1.33)	0.652				1.27	(0.87, 1.86)	0.211			
Age (years)																		
7-8	1.00						1.00						1.00					
9	0.74	(0.46, 1.18)					0.73	(0.45, 1.17)					0.91	(0.56, 1.49)				
10-11	0.79	(0.48, 1.30)	0.440				0.80	(0.48, 1.34)	0.421				0.80	(0.47, 1.36)	0.695			
Socioeconomic status																		
Bottom 40%	1.00						1.00						1.00			1.00		
Middle 40%	1.00	(0.66, 1.51)					0.82	(0.55, 1.24)					0.58	(0.38, 0.90)		0.69	(0.42, 1.12)	
Top 20%	1.22	(0.75, 1.99)	0.680				0.55	(0.32, 0.93)	0.075				0.88	(0.53, 1.46)	0.046	1.06	(0.60, 1.87)	0.223
Weekly meat consumption																		
< 1 times per week	0.61	(0.40, 0.94)	0.022	0.70	(0.45, 1.09)	0.112												
3-4 times per week													0.66	(0.39, 1.12)	0.112	0.76	(0.43, 1.35)	0.125
≥5 times per week							0.57	(0.29, 1.12)	0.084									
Livestock																		
No livestock	0.60	(0.30, 1.19)	0.130	0.51	(0.24, 1.11)	0.072							0.51	(0.24, 1.06)	0.056			
Bullock	1.60	(0.91, 2.83)	0.110	1.63	(0.87, 3.07)	0.131	0.64	(0.32, 1.26)	0.178	0.58	(0.27, 1.24)	0.143	0.58	(0.28, 1.19)	0.121	0.76	(0.35, 1.64)	0.169
Horse/donkey							1.48	(0.99, 2.22)	0.056									
Goat							1.34	(0.89, 2.01)	0.170									
Sheep	0.71	(0.45, 1.14)	0.146				1.61	(1.05, 2.48)	0.030				0.73	(0.46, 1.17)	0.180	0.71	(0.43, 1.18)	0.089
Poultry							1.54	(1.05, 2.25)	0.027	1.43	(0.95, 2.14)	0.089	1.95	(1.31, 2.90)	< 0.001	2.04	(1.34, 3.12)	0.001
Hygiene behaviour																		
Washing hands after defecation with soap																		
Rarely/sometimes	1.00						1.00						1.00					
Often/always	1.14	(0.95, 1.37)	0.160				1.15	(0.96, 1.39)	0.134				1.14	(0.94, 1.39)	0.182			
Eating unpeeled fruits																		
Rarely/sometimes							1.00			1.00								
Often/always							0.83	(0.68, 1.02)	0.073	0.86	(0.69, 1.07)	0.177						
Eating raw vegetables																		
Rarely/sometimes							1.00											
Often/always							1.43	(0.97, 2.12)	0.076									
Wearing sandals outside in summer	0.30	(0.11, 0.84)	0.024															
Wearing closed shoes outside in summer	3.39	(1.12, 10.25)	0.033	3.26	(1.01, 10.43)	0.047												
Sanitation																		
Toilet in household	0.70	(0.47, 1.03)	0.077				0.76	(0.51, 1.14)	0.192									
Toilet in yard	1.47	(0.99, 2.19)	0.060	1.37	(0.88, 2.13)	0.163	1.33	(0.89, 1.98)	0.173	1.39	(0.89, 2.18)	0.149						
Source of drinking water																		
Water tap in yard (shared with neighbours)							1.90	(0.95, 3.81)	0.079									
Public tap/standpipe	0.35	(0.12, 1.00)	0.027	0.27	(0.05, 1.39)	0.094							0.42	(0.16, 1.11)	0.056	0.37	(0.12, 1.13)	0.057
Protected spring							0.52	(0.31, 0.88)	0.010	0.41	(0.23, 0.74)	0.002	0.42	(0.20, 0.88)	0.013	0.44	(0.20, 0.97)	0.020
Surface water: river							1.57	(0.97, 2.52)	0.071									
Surface water: stream							0.35	(0.08, 1.53)	0.113	0.27	(0.06, 1.24)	0.054						
Surface water: river/stream													1.36	(0.86, 2.14)	0.195			

^a ^Crude odds ratio

^b ^School-level random effect included

^c ^*P*-value based on likelihood ratio test (LRT

* Outcome: *G. intestinalis*, *E. histolytica/E*. *dispar *and *H. nana*. Explanatory variables: demographic, socioeconomic, hygiene behaviour and sources of drinking water.

Children belonging to households keeping sheep and poultry were at a slightly higher risk of an *E. histolytica/E. dispar *infection (OR = 1.61, 95% CI: 1.05-2.48; OR = 1.54, 95% CI: 1.05-2.25, respectively). Likewise, *H. nana *infection was associated with chicken farming both in the bi- and multivariate model (OR = 1.95. 95% CI: 1.31-2.90; OR = 2.04, 95% CI: 1.34-3.12). Socioeconomic status was significantly associated with *H. nana *infection, since children from households of the middle 40% were less likely to be infected compared to their poorer counterparts (OR = 0.58, 95% CI: 0.38-0.90).

## Discussion

The present cross-sectional survey determining the prevalence (and intensity) of infection with helminths and intestinal protozoa among 594 children aged 7-11 years in 10 randomly selected schools in western Tajikistan revealed that parasitic infections are a public health issue. Indeed, every third child was infected with helminths and almost every second child harboured at least one intestinal protozoon species. One out of five children had multiple species intestinal parasitic infections. Every fourth child was infected with *H. nana*, *G. intestinalis *and *E. histolytica/E. dispar*. The patterns of intestinal parasitic infections indicated spatial clustering: the school with the highest overall and multiple species infection prevalence showed the highest prevalences of *H. nana*, *A. lumbricoides*, hookworm and *E. histolytica/E. dispar*. Public well/standpipe as drinking water source was found to be a protective factor for *G. intestinalis *infections, whereas protected spring water reduced the risk of infections with *H. nana *and *E. histolytica/E. dispar*. Children from households keeping poultry were more likely to be infected with *H. nana *and *E. histolytica/E. dispar *than children from the remaining households.

The high overall prevalence of intestinal parasites, observed in our study, corroborates previous studies from Central Asia. A population-representative survey in children aged 6-15 years from Kyrgyzstan demonstrated an overall infection prevalence of 41% [7]. Unpublished parasitological data from surveys conducted by the Sanitary Epidemiological Service of Kyrgyzstan among 3, 427 school-aged children in 2006/2007 indicated an overall infection prevalence of 71.4%, with *G. intestinalis *being the most common intestinal protozoon species in that study (23.1%) [34]. Another school-based cross-sectional survey from Afghanistan showed that 47.6% of the subjects were infected with at least one soil-transmitted helminth, predominantly *A. lumbricoides *(40.9%) [35]. According to a recent WHO report, prevalence of soil-transmitted helminth infections in Tajikistan was estimated to range between 20% and 50% [10].

Significant spatial heterogeneity in the prevalence across schools was found, particularly for helminth infections. Lowest prevalences were found in the two schools in the mountainous area. Spatial disparities of infection prevalence were also described from school-based surveys in Haiti [36]. Geographical variation of different soil-transmitted helminths in a study from Zanzibar was interpreted with predominant soil types as a distinguishing factor [37]. Eight schools in our study are located in the ecological zone 'lowland', containing fine-grained alluvial or loessic soils [21] where intensive irrigated agriculture is practiced [38]. The observed clustering of intestinal parasites (i.e. *H. nana*, *A. lumbricoides*, hookworm and *E. histolytica/E. dispar*) in one of the investigated schools corroborates findings of small-scale clustering (e.g. household level). *A. lumbricoides *and *T. trichiura *were observed to aggregate at household level in a cross-sectional survey conducted in the People's Republic of China [39]. Another study, conducted in rural Amazonian settlements, observed that almost half of the helminth infections were concentrated in only 5% of the surveyed households [40]. Some authors differentiated between domestic (household area) and public transmission sites (public places of work, streets, fields and schools) and recommended that control measures should target both domains [41].

**Figure 2:**
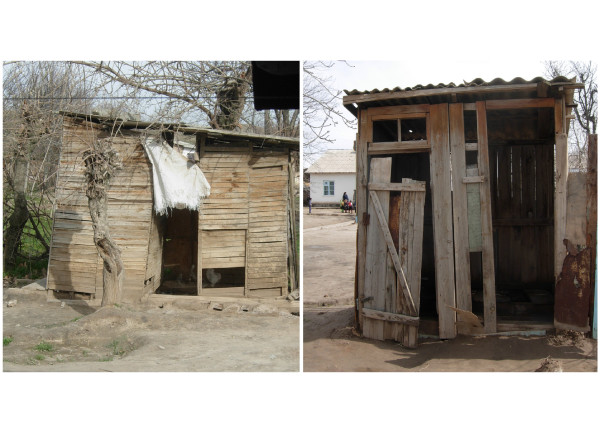
**School latrines in two primary schools in western Tajikistan, early 2009**.

Nearly half of all drinking water sources reported by the children in the current study were classified as unimproved sources, but a large variation was found (2-100% at the unit of the school). Teachers explained that electricity in their villages is often unstable and available only for a few hours per day, particularly during the winter season. When community water supply systems operated by electric pumps are interrupted, people draw water for domestic needs from open and unprotected sources such as irrigation canals and rivers. A large part of the latrines in the schools visited were inappropriately maintained (Figure 2).

Our findings underline UNICEF estimations from 2007; almost half of the rural households in Tajikistan depended on unimproved drinking water sources [42]. Unmet drinking water and sanitation standards in Tajikistan partially result from weak services of water supply and public sanitation. Only 23% of the population had access to a sewage system in 2003; 89% in urban areas but only 11% in rural areas [6]. Thus far, active participation mechanisms in water management involving public and private sectors and local communities are poorly developed [6,43].

Interestingly, the use of unimproved drinking water sources did not emerge as a risk factor for *G. intestinalis *and *E. histolytica/E. dispar *infection in our study. In other settings, however, water sources were identified as a risk factor for *Giardia*, as this intestinal protozoon species is commonly transmitted by ingesting cysts persisting in contaminated water or from person-to-person through the faecal-oral route [4]. Our study indicates that the use of drinking water from improved sources (public well/standpipe) is a protective factor for infections with *E. histolytica/E. dispar*, *G. intestinalis *and *H. nana*. Similar findings were observed for *H. nana *from Kyrgyzstan [7]. The use of tap water was reported to be associated with low infection prevalence of *G. intestinalis *compared to the use of surface water in a school-based survey in Côte d'Ivoire [44]. A study from Mexico City identified the storing of drinking water in unprotected containers (cisterns, tanks and bucks) as a risk factor for *G. intestinalis *[45].

Our study has some limitations. First, only one stool sample was collected from each participant. Previous research has shown that multiple stool sampling enhances the sensitivity of helminths and intestinal protozoa diagnosis [46,47]. Second, the Kato-Katz technique is inappropriate for accurate diagnosis of *E. vermicularis *and *Strongyloides stercoralis*. Indeed, the adhesive tape method is recommended for *E. vermicularis *diagnosis, but there are compliance issues with this method [33]. For *S. steroralis *diagnosis, the Baerman and/or the Koga agar plate method should be used [48]. To partially overcoming these shortcomings, we prepared duplicate Kato-Katz thick smears and preserved 1-2 g of stool that was subjected to an additional diagnostic approach, the ether-concentration method. Data from both methods combined were considered as diagnostic 'gold' standard. Third, no attempt was made to investigate seasonality. We speculate that the prevalence of parasitic infections might be higher in summer when children spend more time outside and might eat more frequently unwashed vegetables and fruits from the garden, as has been observed in neighbouring Kyrgyzstan [49].

## Conclusions

The present study provides new insight into school-aged children's infection status with helminths and intestinal protozoa in ecological 'lowland' areas of western Tajikistan. Considering the high infection prevalence of *H. nana*, *E. histolytica/E. dispar *and *G. intestinalis *observed here, a way forward may consist in locally adapted interventions, combining an initial school-based deworming and targeted health education programmes, promoting better hygiene and improved sanitation. Treatment with albendazole is proposed to control soil-transmitted helminthiasis, whereas metronidazole should be utilized against the two pathogenic intestinal protozoa. Previous research has shown that carefully designed school-based hygiene programmes effectively contributed to reduce infection intensity and re-infection rates [49-52]. A nationwide deworming programme in Tajikistan is currently conceived by the MoH and the RTDC. In 2010, a total of 32 laboratory technicians received refresher training on specific laboratory diagnostic techniques for identification of soil-transmitted helminths. In our view, further investigations are warranted to assess the true public health burden due to *H. nana *infection to guide future control efforts against this helminth, which represented the predominant species in our study area.

## Competing interests

The authors declare that they have no competing interests.

## Authors' contributions

BM, JU and KW conceived and designed the study protocol, data collection forms and questionnaires for interviews. BM and GK planned, coordinated and supervised data collection in the field. GK, ZM and VJR conducted interviews and performed data collection. MB supervised the collection and analysis of parasitological data. MH, MK and LKL provided laboratory analyses of stool samples. Data analysis and writing up of the manuscript was done by BM. BM, JU and KW revised the manuscript. The final version of the manuscript was reviewed and approved by all authors prior to submission.
